# Supplementary material for: How coping styles moderate the relationship between diabetes distress and self-care in adults with diabetes in Appalachian Ohio: A cross-sectional survey study
Source: PLoS One. 2025 Dec 2;20(12):e0337481. doi: 10.1371/journal.pone.0337481 (PMC12671808; doi:10.1371/journal.pone.0337481)
Supplement: S1 Table — Legend: Diabetes Distress Total Scores = Type 1 Diabetes Distress Scale and Subscales and Type 2 Diabetes Distress Scale and Subscales; Depressive Symptoms = Patient Health Questionnaire-9 (PHQ-9); Diabetes Self-Care = Self-Care Inventory-Revised; Problem-Based Coping and Emotion-Based Coping = Coping Styles; Diabetes Self-Efficacy = Confidence in Diabetes Self-Care Scale; Social Support = Medical Outcomes Study Social Support Survey. (DOCX) [file pone.0337481.s005.docx]

S1 Table. Means and Standard Deviations of Psychosocial Measures among Participants with Type 1 and Type 2 Diabetes (n=256)

|  | Type 1 Diabetes  n=99 | Type 2 Diabetes  n=157 |  |  |
| --- | --- | --- | --- | --- |
| Variable¤ | Mean ± SD | Mean ± SD | t-value | p |
| Diabetes Distress Total Scores^a^¤ | 2.4±1.0¤ | 2.4±1.0¤ | 0.194¤ | 0.847¤ |
| ^→^Powerlessness Subscale^a^¤ | 3.1±1.2¤ | - | - | - |
| ^→^Management Distress Subscale^a^¤ | 2.5±1.3¤ | - | - | - |
| ^→^Hypoglycemia Distress Subscale^a^¤ | 2.3±1.1¤ | - | - | - |
| ^→^Negative Social Perceptions Subscale^a^¤ | 2.2±1.1¤ | - | - | - |
| ^→^Eating Distress Subscale^a^¤ | 2.6±1.3¤ | - | - | - |
| ^→^Physician Distress Subscale^a^¤ | 2.0±1.3¤ | - | - | - |
| ^→^Friend/Family Distress Subscale^a^¤ | 2.3±1.2¤ | - | - | - |
| ^→^Emotional Burden Subscale^a^¤ | - | 2.7±1.4¤ | - | - |
| ^→^Physician Distress Subscale^a^¤ | - | 1.6±1.1¤ | - | - |
| ^→^Regimen Distress Subscale^a^¤ | - | 2.8±1.3¤ | - | - |
| ^→^Interpersonal Distress Subscale^a^¤ | - | 2.2±1.3¤ | - | - |
| Depressive Symptoms^b^¤ | 4.3±5.4¤ | 5.4±6.0¤ | -1.487¤ | 0.138¤ |
| Diabetes Self-Care^c^¤ | 4.1±0.9¤ | 3.0±0.7¤ | 5.347¤ | <0.001¤ |
| Problem-Based Coping^d^¤ | 2.6±0.4¤ | 2.5±0.5¤ | 1.447¤ | 0.149¤ |
| Emotion-Based Coping^d^¤ | 2.3±0.6¤ | 2.3±0.7¤ | 0.615¤ | 0.539¤ |
| eDiabetes Self-Efficacy^e^¤ | 73.8±21.5¤ | 72.0±18.7¤ | 0.709¤ | 0.479¤ |
| Social Support^f^¤ | 4.1±0.9¤ | 3.8±1.1¤ | 2.716¤ | 0.007¤ |

^a^Diabetes Distress Total Scores=Type 1 Diabetes Distress Scale and Subscales and Type 2 Diabetes Distress Scale and Subscales; ^b^Depressive Symptoms=Patient Health Questionnaire-9 (PHQ-9); ^c^Diabetes Self-Care=Self-Care Inventory-Revised; ^d^Problem-Based Coping and Emotion-Based Coping=Coping Styles; ^e^Diabetes Self-Efficacy=Confidence in Diabetes Self-Care Scale; ^f^Social Support=Medical Outcomes Study Social Support Survey
