# Supplementary material for: How coping styles moderate the relationship between diabetes distress and self-care in adults with diabetes in Appalachian Ohio: A cross-sectional survey study
Source: PLoS One. 2025 Dec 2;20(12):e0337481. doi: 10.1371/journal.pone.0337481 (PMC12671808; doi:10.1371/journal.pone.0337481)
Supplement: Attachment — Submitted filename: PONE-D-25-08613.R1_Responses to Reviewer Comments.docx [file pone.0337481.s006.docx]

PONE-D-25-08613.R1

We sincerely thank the editor and reviewers for their thoughtful and constructive feedback. We carefully considered each comment and made corresponding revisions throughout the manuscript. Your insights have significantly strengthened the clarity, rigor, and overall quality of our paper.

Journal requirements:

When submitting your revision, we need you to address these additional requirements.

1.  Please ensure that your manuscript meets PLOS ONE's style requirements, including those for file naming. The PLOS ONE style templates can be found at

[https://journals.plos.org/plosone/s/file?id=wjVg/PLOSOne_formatting_sample_main_body.pdf](https://nam11.safelinks.protection.outlook.com/?url=https%3A%2F%2Ftrack.editorialmanager.com%2FCL0%2Fhttps%3A%252F%252Fjournals.plos.org%252Fplosone%252Fs%252Ffile%253Fid%3DwjVg%252FPLOSOne_formatting_sample_main_body.pdf%2F1%2F010f0196c3fa7fa8-4c75cd0e-8f0d-4d07-9d22-7bd4e537d288-000000%2Fcqq69iHV5HjgLJDhsvLJkgIkfcRGe2qypRkeVO5yvDY%3D210&data=05%7C02%7Cbeverle1%40ohio.edu%7Cd65136a21c764d920e8408dd913d614e%7Cf3308007477c4a70888934611817c55a%7C0%7C0%7C638826415304376399%7CUnknown%7CTWFpbGZsb3d8eyJFbXB0eU1hcGkiOnRydWUsIlYiOiIwLjAuMDAwMCIsIlAiOiJXaW4zMiIsIkFOIjoiTWFpbCIsIldUIjoyfQ%3D%3D%7C0%7C%7C%7C&sdata=Cm4SoRSqOFwhh9f%2BQhgPwdS19dhXisgIv%2FvierGlLN0%3D&reserved=0)  and

[https://journals.plos.org/plosone/s/file?id=ba62/PLOSOne_formatting_sample_title_authors_affiliations.pdf](https://nam11.safelinks.protection.outlook.com/?url=https%3A%2F%2Ftrack.editorialmanager.com%2FCL0%2Fhttps%3A%252F%252Fjournals.plos.org%252Fplosone%252Fs%252Ffile%253Fid%3Dba62%252FPLOSOne_formatting_sample_title_authors_affiliations.pdf%2F1%2F010f0196c3fa7fa8-4c75cd0e-8f0d-4d07-9d22-7bd4e537d288-000000%2FkqbXzZy0IThyI2616553PP43bJ2y10xrzYRdJ9BwuK4%3D210&data=05%7C02%7Cbeverle1%40ohio.edu%7Cd65136a21c764d920e8408dd913d614e%7Cf3308007477c4a70888934611817c55a%7C0%7C0%7C638826415304385540%7CUnknown%7CTWFpbGZsb3d8eyJFbXB0eU1hcGkiOnRydWUsIlYiOiIwLjAuMDAwMCIsIlAiOiJXaW4zMiIsIkFOIjoiTWFpbCIsIldUIjoyfQ%3D%3D%7C0%7C%7C%7C&sdata=g6%2FO5Ub6ryNI5nu4%2F9LqaWklXn%2FB15gallYC%2B1OTzKs%3D&reserved=0)

Response: Thank you for sharing the style templates. We have reviewed the formatting and made revisions to adhere to the style requirements and file naming.

2. Thank you for stating the following financial disclosure:

“American Osteopathic Association Grant: Osteopathic Approach to Diabetes: Pathophysiology, Treatment, Outcomes and Complication Grant (No. 1291708718)”

Please state what role the funders took in the study.  If the funders had no role, please state: "The funders had no role in study design, data collection and analysis, decision to publish, or preparation of the manuscript."

If this statement is not correct you must amend it as needed.

Please include this amended Role of Funder statement in your cover letter; we will change the online submission form on your behalf.

Response: Thank you. This is correct. The funder had no role in the study design, data collection and analysis, decision to publish, or preparation of the manuscript. We have included this in the cover letter.

3. In the online submission form, you indicated that [All relevant data are within the manuscript and supporting tables. The dataset underlying the results presented in the study are available upon request from the corresponding author.].

All PLOS journals now require all data underlying the findings described in their manuscript to be freely available to other researchers, either 1. In a public repository, 2. Within the manuscript itself, or 3. Uploaded as supplementary information.

This policy applies to all data except where public deposition would breach compliance with the protocol approved by your research ethics board. If your data cannot be made publicly available for ethical or legal reasons (e.g., public availability would compromise patient privacy), please explain your reasons on resubmission and your exemption request will be escalated for approval.

Response: We have uploaded all relevant data to a public repository. The dataset is in Mendeley. Here in the information for the public repository: Beverly, Elizabeth (2025), “EBeverlyDiabetesDistressCopingDataset”, Mendeley Data, V1, doi: 10.17632/rrx6stxcfk.1 or <https://data.mendeley.com/datasets/rrx6stxcfk/1>

4. When completing the data availability statement of the submission form, you indicated that you will make your data available on acceptance. We strongly recommend all authors decide on a data sharing plan before acceptance, as the process can be lengthy and hold up publication timelines. Please note that, though access restrictions are acceptable now, your entire data will need to be made freely accessible if your manuscript is accepted for publication. This policy applies to all data except where public deposition would breach compliance with the protocol approved by your research ethics board. If you are unable to adhere to our open data policy, please kindly revise your statement to explain your reasoning and we will seek the editor's input on an exemption. Please be assured that, once you have provided your new statement, the assessment of your exemption will not hold up the peer review process.

Response: Thank you for this comment. We have uploaded all relevant data to a public repository. The dataset is in Mendeley. Here in the information for the public repository: Beverly, Elizabeth (2025), “EBeverlyDiabetesDistressCopingDataset”, Mendeley Data, V1, doi: 10.17632/rrx6stxcfk.1 or <https://data.mendeley.com/datasets/rrx6stxcfk/1>

5. Please review your reference list to ensure that it is complete and correct. If you have cited papers that have been retracted, please include the rationale for doing so in the manuscript text, or remove these references and replace them with relevant current references. Any changes to the reference list should be mentioned in the rebuttal letter that accompanies your revised manuscript. If you need to cite a retracted article, indicate the article’s retracted status in the References list and also include a citation and full reference for the retraction notice.

Response: We have looked up every article on our reference list to see if any articles were retracted. Our reference list is complete and correct. None of our cited articles have been retracted. We have two articles listed with Erratums (Young-Hyman D et al., 2016; Ryan D et al., 2020). Additionally, we have three articles that are not indexed in PubMed (Ngan et al., 2023; Pearson et al., 2018; Schroevers et al., 2015). We included those articles because they were mindfulness based randomized controlled trials and an acceptance-based diabetes education randomized controlled trial, which are pertinent to diabetes distress.

 Additional Editor Comments:

Thanks for submitting a well-written manuscript that covers a very relevant subject. However, the manuscript requires a few minor revisions as suggested by the expert reviewers before it can be taken into consideration. Taking into account changes could undoubtedly enhance the manuscript's readability and quality.

[Note: HTML markup is below. Please do not edit.]

Reviewers' comments:

Reviewer's Responses to Questions

**Comments to the Author**

1. Is the manuscript technically sound, and do the data support the conclusions?

The manuscript must describe a technically sound piece of scientific research with data that supports the conclusions. Experiments must have been conducted rigorously, with appropriate controls, replication, and sample sizes. The conclusions must be drawn appropriately based on the data presented.

Reviewer #1: Yes

Reviewer #2: Partly

2. Has the statistical analysis been performed appropriately and rigorously?

Reviewer #1: Yes

Reviewer #2: No

Response: We have revised all statistical analyses. We believe they have been performed appropriately and rigorously in this revision.

3. Have the authors made all data underlying the findings in their manuscript fully available?

The [PLOS Data policy](https://nam11.safelinks.protection.outlook.com/?url=http%3A%2F%2Ftrack.editorialmanager.com%2FCL0%2Fhttp%3A%252F%252Fwww.plosone.org%252Fstatic%252Fpolicies.action%2523sharing%2F1%2F010f0196c3fa7fa8-4c75cd0e-8f0d-4d07-9d22-7bd4e537d288-000000%2FHhs0X-FXK46AyBE76bnt6MpH_dVBLzgfDrgbiMdijAs%3D210&data=05%7C02%7Cbeverle1%40ohio.edu%7Cd65136a21c764d920e8408dd913d614e%7Cf3308007477c4a70888934611817c55a%7C0%7C0%7C638826415304394866%7CUnknown%7CTWFpbGZsb3d8eyJFbXB0eU1hcGkiOnRydWUsIlYiOiIwLjAuMDAwMCIsIlAiOiJXaW4zMiIsIkFOIjoiTWFpbCIsIldUIjoyfQ%3D%3D%7C0%7C%7C%7C&sdata=as7v%2FEsvdejB6PaZTW1bNuWIBin3KbI5ng75KNLoAs0%3D&reserved=0) requires authors to make all data underlying the findings described in their manuscript fully available without restriction, with rare exception (please refer to the Data Availability Statement in the manuscript PDF file). The data should be provided as part of the manuscript or its supporting information, or deposited to a public repository. For example, in addition to summary statistics, the data points behind means, medians and variance measures should be available. If there are restrictions on publicly sharing data—e.g. participant privacy or use of data from a third party—those must be specified.

Reviewer #1: Yes

Reviewer #2: No

Response: We have uploaded all relevant data to a public repository. The dataset is in Mendeley. Here in the information for the public repository: Beverly, Elizabeth (2025), “EBeverlyDiabetesDistressCopingDataset”, Mendeley Data, V1, doi: 10.17632/rrx6stxcfk.1 or <https://data.mendeley.com/datasets/rrx6stxcfk/1>

4. Is the manuscript presented in an intelligible fashion and written in standard English?

PLOS ONE does not copyedit accepted manuscripts, so the language in submitted articles must be clear, correct, and unambiguous. Any typographical or grammatical errors should be corrected at revision, so please note any specific errors here.

Reviewer #1: Yes

Reviewer #2: Yes

5. Review Comments to the Author

Please use the space provided to explain your answers to the questions above. You may also include additional comments for the author, including concerns about dual publication, research ethics, or publication ethics. (Please upload your review as an attachment if it exceeds 20,000 characters)

Reviewer #1: This manuscript addresses a highly relevant and timely topic: the interaction between coping styles and diabetes distress in relation to self-care behaviors among adults with type 1 and type 2 diabetes. The study design is appropriate, and the sample size is adequate. The use of validated tools enhances the credibility of the findings.

Response: Thank you for the positive feedback.

Strengths:

Clear and justified research questions and hypotheses.

Robust statistical methods with a clear rationale for interaction testing.

Important clinical implications for diabetes education and behavioral interventions.

Well-articulated discussion connecting findings to existing literature and practice.

Response: Thank you for the positive feedback.

Areas for Minor Improvement:

Clarify Missing Data: While the manuscript acknowledges some missing values in demographics and lab measures, it would help to elaborate briefly on how missing data were handled in the regression models.

Response: Thank you for asking this question. For this revision, we were asked to conduct a multi-step data verification process to ensure accurate classification of participants with type 1 and type 2 diabetes. Our process included sequential checks for medication congruence, scale completion, consistency between age and diabetes duration, plausible A1C values, and duplicate entries prior to final analyses (see Supplemental Figure). As a result, we excluded 69 cases from our previous sample size (n=31 type 1 diabetes, n=38 with type 2 diabetes). The removal of these cases also removed the majority of cases with missing data. In our regression models, the only variable with missing data is age (n=1).

Figure/Tables Enhancements: If space permits, consider including a simple interaction plot showing the moderating effect of problem-focused coping on the diabetes distress-self-care relationship.

Response: Thank you for this comment. We have included three interaction plots as supplemental information to show the moderating effect of problem-based coping and emotion-based coping on the diabetes distress-self-care relationship.

Language/Typo: In the Abstract, line 32 – "This purpose of this study" should be corrected to "The purpose of this study".

Response: Thank you for noting this typo. We have corrected it in the Abstract.

Limitations: The limitations section is candid and well-written, though it may benefit from explicitly noting the potential for self-selection bias due to voluntary survey participation.

Response: Thank you. We have added self-selection as a limitation to our study.

Data Transparency: Although the data availability is noted, the authors may consider adding a brief supplemental table with mean scores and standard deviations for each major construct (e.g., coping, self-care), to facilitate understanding and reproducibility.

Response: Excellent suggestion. We have added this table as supplemental information. In addition, we have uploaded our dataset to a publicly available registry.

Overall, this is a valuable contribution to the literature on psychosocial factors in diabetes management. With some minor revisions, it is suitable for publication.

Response: Thank you for the kind feedback.

Reviewer #2: Summary:

The study evaluates effects of degree of diabetes distress and coping strategies on diabetes self-care, measured by questionnaires in an arbitrary selection of Ohio residents. The study is interesting, although novelty needs to be clarified. Furthermore, T1D and T2D should be separated as they are two distinctly different diseases.

Response: Thank you for this comment. We have revised our analyses and conducted separate models for type 1 and type 2 diabetes. In addition, we clarified that this study was a confirmation study in a unique population of Appalachian Ohio.

Major:

1. The participant recruitment could be clarified. Where were participants recruited specifically and who were the advertisements targeting and where were flyers distributed, in specific communities or cities etc? What measures were taken to ascertain a sample representative of the overall Ohio population?

Response: Thank you for this comment. We have included additional information about recruitment. This study focuses on southeastern Ohio, which is a seven-county region in southeastern Appalachian Ohio: “Recruitment emails were distributed to all employees at the University, which is the largest employer in the seven-county region, and to individuals listed in the Diabetes Institute registry who had previously given permission to be contacted for future research opportunities. In addition, study flyers were posted in public locations, including grocery stores, libraries, primary care offices, and the seven country health departments. Finally, written advertisements were printed in the local newspaper and broadcast on the local public radio station to increase recruitment reach and participation across the seven-county region.”

2. Please include a step-by-step flow chart of all steps taken to determine if a participant had or did not have a diabetes diagnosis as supplementary material. Each step should include the n at that stage to illustrate how many are lost in the process. There is both a risk of defining the wrong individuals, but perhaps even more so, to define individuals with diabetes as non-diabetics due to insufficient reporting. This may affect the representativity of the study as participants with low insight may be missed.

Response: Thank you for this recommendation. We conducted a multi-step data verification process to ensure accurate classification of participants with type 1 and type 2 diabetes. Our process included sequential checks for medication congruence, scale completion, consistency between age and diabetes duration, plausible A1C values, and duplicate entries prior to final analyses (see Supplemental Figure). As a result, we excluded 69 cases from our previous sample size (n=31 type 1 diabetes, n=38 with type 2 diabetes). The removal of these cases also removed most cases with missing data. We believe this multi-step process determined whether or not a person had diabetes and which type of diabetes.

3. Please separate individuals with T1D and T2D in this study (all data analysis). They are two completely different diseases, with different treatments, etiology and health risks. Furthermore, it is not appropriate to combine two different scoring systems into one (diabetes distress in T1D and T2D) as this will bias the regression analysis.

Response: Thank you for this comment. We have separated the analyses and the models. All data are presented separately.

4. Please specify the primary outcome of the study in the statistics section. The title currently suggests that coping is an exposure and distress is the outcome. This makes sense but is not currently what is tested in the regression model (where distress and coping are both exposures.

Response: We have clarified the primary outcome in the statistics section as well as in the title. Thank you.

5. As discussed there are multiple intervention studies targeting diabetes distress, but it is not clearly communicated to me what new information this cross-sectional study contributes with in relation to what has already been demonstrated in human interventional experiments for T1D and T2D respectively. While it is suggested on line 337 what direction to take going forward, it appears to me that several studies already discussed have already successfully done what is suggested by the authors? Perhaps this study is more a confirmatory study, indicating that previous successful intervention studies may also hold feasibility for residents in Ohio?

Response: We agree that this is not a new finding, and previous intervention studies have shown that addressing coping strategies can reduce diabetes distress. The purpose of this paper was to replicate and confirm prior research and contribute to the literature on this topic. Furthermore, we aimed to replicate and confirm this research in a unique population, rural Appalachian Ohio. We have added information in our Introduction and Methods explaining the uniqueness of the region and culture.

Minor:
1. Are “Indian” and “race” adequate terminology? Consider eg. native Americans and ethnicity as alternative terms.

Response: We followed the NIH reporting of race terminology, which includes American Indian or Alaska Native, Asian, Black or African American, Native Hawaiian or Other Pacific Islander, White (see <https://www.nih.gov/nih-style-guide/race-national-origin>)

2. 9.5% of participants have a doctoral degree, is this representative of residents of Ohio? Please discuss.

Response: Thank you for making this point. In our revised sample, 9.4% of participants had a Master’s degree and 10.5% of participants had a doctoral degree or professional degree. In 2024, 12.5% of people in Ohio had a graduate or professional degree. Our sample is higher than the state average. We attribute this to recruiting faculty, administration, and staff from the university. We have acknowledged this in our Limitations.

3. Approx 1/4 with T2D appear to be on insulin, is this representative of the Ohio population, it appears to be a high proportion in relation to current guidelines. What does this say about sample selection? Please discuss.

Response: For this revision, we separated the analyses and included a revised table of demographic and health characteristic data. The percentage of participants with type 2 diabetes on insulin only is 8.9% (n=14) and the percentage of participants with type 2 diabetes on insulin and oral medications is 24.2% (n=38). Recent nationally representative data on insulin use prevalence is relatively scare for the type 2 diabetes population. Older studies have reported rates of insulin use ranging from 22% to 36% in the type 2 diabetes population (with most insulins being long-acting as in our study). We believe that our revised data are representative of the Ohio and U.S. population.

4. Clarify gender and occupation in table 2 (what does low/high mean). Is duration diabetes duration?

Response: With our revised analyses, we have removed gender and occupation from the correlation table. Yes, duration is diabetes duration. We have made that correction to the table.

5. Please elaborate on the coping style questionnaire, what is the outcome? Are participants separated in either type or do you ge a final score of up to 60 points (15*4), where low is emotional and high is pragmatic?

Response: Great question. We have provided a more detailed explanation of the Coping Style questionnaire in the Methods section.

6. What are the cutoffs for moderate and high diabetes distress, add to methods with reference.

Response: Thank you. For the Diabetes Distress Scale, we used the cut-points are little or no distress=<2.0; moderate distress=2.0–2.9; high distress=≥3.0 from Fisher L et al. Diabetes Care. 2012 Jan 16;35(2):259–264. doi: [10.2337/dc11-1572](https://doi.org/10.2337/dc11-1572) We have included this in the Methods. For the Type 1 Diabetes Distress Scale, we little or no distress (1.0–1.4), mild distress (1.5–1.9), moderate distress (2.0–2.9), and high distress (≥ 3.0) from Fisher L et al. J Diabetes Complications. 2015 Feb 7;29(4):572–577. doi: [10.1016/j.jdiacomp.2015.01.012](https://doi.org/10.1016/j.jdiacomp.2015.01.012).

7. Please clarify how you handled non-normally distributed variables as A1c. The outliers around >100 mmol/mol will likely pivot alla analysis and a sensitivity analysis should exclude these individuals to validate current associations found for A1c.

Response: Thank you for this comment. We plotted our A1cs and used the Kolmogorov-Smirnov statistic to determine that the A1cs were distributed non-normally. Next, we applied a base-10 logarithmic transformation to A1c values to reduce skewness and improve the linearity of relationships between A1c and other variables.

8. Consider if z-transformation of reported scales may be more appropriate to include in linear regression models than untransformed scales.

Response: Thank you for this recommendation. We did the z-transformation for all of the scales included in the regression models.

6. PLOS authors have the option to publish the peer review history of their article ([what does this mean?](https://nam11.safelinks.protection.outlook.com/?url=https%3A%2F%2Ftrack.editorialmanager.com%2FCL0%2Fhttps%3A%252F%252Fjournals.plos.org%252Fplosone%252Fs%252Feditorial-and-peer-review-process%2523loc-peer-review-history%2F1%2F010f0196c3fa7fa8-4c75cd0e-8f0d-4d07-9d22-7bd4e537d288-000000%2Fs4lXcz2lvYO_qgsJFBQedp0sZFvlcEhHPUQpkQxSD8M%3D210&data=05%7C02%7Cbeverle1%40ohio.edu%7Cd65136a21c764d920e8408dd913d614e%7Cf3308007477c4a70888934611817c55a%7C0%7C0%7C638826415304404199%7CUnknown%7CTWFpbGZsb3d8eyJFbXB0eU1hcGkiOnRydWUsIlYiOiIwLjAuMDAwMCIsIlAiOiJXaW4zMiIsIkFOIjoiTWFpbCIsIldUIjoyfQ%3D%3D%7C0%7C%7C%7C&sdata=5lLTbO%2B7%2BgZ2Y4%2FEow%2FY5h%2FVSZeqvjp5rHSsVZFZasc%3D&reserved=0)). If published, this will include your full peer review and any attached files.

If you choose “no”, your identity will remain anonymous but your review may still be made public.

**Do you want your identity to be public for this peer review?** For information about this choice, including consent withdrawal, please see our [Privacy Policy](https://nam11.safelinks.protection.outlook.com/?url=https%3A%2F%2Ftrack.editorialmanager.com%2FCL0%2Fhttps%3A%252F%252Fwww.plos.org%252Fprivacy-policy%2F1%2F010f0196c3fa7fa8-4c75cd0e-8f0d-4d07-9d22-7bd4e537d288-000000%2FZzS_BSfutrw7BFj2sRqR91ZSAzWg0EU3bE5b3_umoTw%3D210&data=05%7C02%7Cbeverle1%40ohio.edu%7Cd65136a21c764d920e8408dd913d614e%7Cf3308007477c4a70888934611817c55a%7C0%7C0%7C638826415304413322%7CUnknown%7CTWFpbGZsb3d8eyJFbXB0eU1hcGkiOnRydWUsIlYiOiIwLjAuMDAwMCIsIlAiOiJXaW4zMiIsIkFOIjoiTWFpbCIsIldUIjoyfQ%3D%3D%7C0%7C%7C%7C&sdata=BZhQUWX8o2%2BzidGFYhPZGLMvFL0eN%2BxjNzb96EMhxb4%3D&reserved=0).

Reviewer #1: **Yes:** Elabbass Ali Abdelmahmuod

Reviewer #2: No

[NOTE: If reviewer comments were submitted as an attachment file, they will be attached to this email and accessible via the submission site. Please log into your account, locate the manuscript record, and check for the action link "View Attachments". If this link does not appear, there are no attachment files.]

While revising your submission, please upload your figure files to the Preflight Analysis and Conversion Engine (PACE) digital diagnostic tool, [https://pacev2.apexcovantage.com/](https://nam11.safelinks.protection.outlook.com/?url=https%3A%2F%2Ftrack.editorialmanager.com%2FCL0%2Fhttps%3A%252F%252Fpacev2.apexcovantage.com%252F%2F1%2F010f0196c3fa7fa8-4c75cd0e-8f0d-4d07-9d22-7bd4e537d288-000000%2FfKGJ5y5owhBpFCQlOoDfDo1-njZrRRth6pKmHWnmDzE%3D210&data=05%7C02%7Cbeverle1%40ohio.edu%7Cd65136a21c764d920e8408dd913d614e%7Cf3308007477c4a70888934611817c55a%7C0%7C0%7C638826415304422194%7CUnknown%7CTWFpbGZsb3d8eyJFbXB0eU1hcGkiOnRydWUsIlYiOiIwLjAuMDAwMCIsIlAiOiJXaW4zMiIsIkFOIjoiTWFpbCIsIldUIjoyfQ%3D%3D%7C0%7C%7C%7C&sdata=DXJVSYARIgpkgjGVRIKhQR5fL1RD17VxLZruW2a2Imk%3D&reserved=0). PACE helps ensure that figures meet PLOS requirements. To use PACE, you must first register as a user. Registration is free. Then, login and navigate to the UPLOAD tab, where you will find detailed instructions on how to use the tool. If you encounter any issues or have any questions when using PACE, please email PLOS at [figures@plos.org](mailto:figures@plos.org). Please note that Supporting Information files do not need this step.
